# Pomological and Molecular Characterization of Pear Cultivars in the German Fruit Genebank

**DOI:** 10.3390/plants15213385

**Published:** 2026-11-01

**Authors:** Lea Broschewitz, Henryk Flachowsky, Erik Schulte, Stefanie Reim, Monika Höfer

**Affiliations:** 1 Julius Kühn Institute (JKI)—Federal Research Centre for Cultivated Plants, Institute for Breeding Research on Fruit Crops, Pillnitzer Platz 3a, 01326 Dresden, Germany; leabroschewitz@gmail.com (L.B.); stefanie.reim@julius-kuehn.de (S.R.); monika.hoefer@julius-kuehn.de (M.H.); 2 Federal Plant Variety Office, Testing Station Wurzen, Torgauer Straße 100, 04808 Wurzen, Germany; erik.schulte@bundessortenamt.de

**Keywords:** genetic structure, parentage analysis, genetic relationships, genetic resources

## Abstract

Pears (*Pyrus* spp.) are an important fruit crop worldwide. In Germany, the German Fruit Genebank manages a large collection of pear cultivars. In this work, we examined 421 pear cultivars of the German Fruit Genebank with regard to cultivar authenticity. *Pyrus* UNiQue genotype codes (PUNQs) were used for international harmonization of the genotype data. More than 60% of the cultivars have accessions which are true-to-type or true-to-type with reservation. We showed that a subset of SSR markers is suitable to distinguish all cultivars within the given dataset. An extensive parentage analysis unravelled 19 candidate offspring-parent trios. An investigation of the genetic structure revealed weak correlation between genetic structure and cultivar origin. In a tree based on genetic relationships, the conclusions of the previous two analysis were combined. We could observe sub-branches based on cultivar origin country and in relation to specific breeding programmes and parentage. Overall, we presented the value of the German Fruit Genebank’s pear collection and suitable analyses revealed new insights in the genetic structure and parentage.

## 1. Introduction/Background

On the world market, Europe produces almost 15% of pear (*Pyrus* spp.) crop, while Asia clearly takes the lead with 73% [1]. In Germany, pear is a staple in the top ten of locally produced fruit but yield is remarkably behind apple production and in constant competition with sweet cherry, plums and damsons [2]. The pear production is dominated by prominent cultivars such as ‘Conference’, ‘Alexander Lucas’, ‘Williams Christ’ and ‘Nojabrskaja’ (Xenia^®^) [3]. Worldwide, China is the leading producer of pears with the focus on the cultivar ‘Ya Li’ [4].

Pear cultivation in Europe has a long-standing history, especially between the 16th and 19th century when the diversity of cultivars increased enormously [4]. Crossbred breeding of pears in Germany began in the 19th century, but today, there are only a few active programmes maintained, for example, at the Julius Kühn Institute, Federal Research Centre for Cultivated Plants, Institute for Breeding Research on Fruit Crops (Dresden-Pillnitz, pome fruit breeder Dr. Andreas Peil) or Bayerisches Obstzentrum GmbH & Co. KG (Hallbergmoos, managing director Dr. Michael Neumüller). Mentionable cultivars that were patented in Germany are, e.g., the Saxonia^TM^ cultivars [4] or the cultivar Alessia^®^ [5]. Internationally, there are pear breeding programmes in, e.g., Switzerland [6], Belgium [7] and the USA [8]. There is a pressing need to keep up with the demand by establishing sufficient pear production. Yet stable production is threatened by the changing climate and diseases, and breeding will possibly become relevant in the future again to tackle these challenges.

The management of genetic resources is the key for future crop improvement, as it provides access to underused and undiscovered genetic material. Here, it is important that the plant genetic material is not only preserved but also examined and studied. For example, Japanese scientists published extensive studies on their pear breeding advances [9], parentage analysis [10], and kinship and inbreeding estimations [11]. Korean scientists used SSR marker analysis to investigate parentages and self-incompatibility [12,13]. The USDA-ARS National *Pyrus* Collection includes many more pear species that have been used to conduct a complex pedigree reconstruction [14]. In Europe, pear germplasm collections were examined in, e.g., Italy [15], Portugal [16] and Spain [17], which are among the main pear producers.

In Germany, the German Fruit Genebank (GFG; https://www.deutsche-genbank-obst.de/ accessed on 4 July 2026) manages and conserves fruit crop genetic resources [18]. The GFG is a national decentralized network that provides fruit genetic resources for breeding and research through long-term preservation [19]. For different indigenous fruit species, there are individual partner networks (e.g., apple network, cherry network, pear network) established [20]. The work presented here is based on the plant material of the GFG’s pear network. The criteria for relevant cultivar conservation in the GFG networks are “(1) German cultivars including new German selections, (2) cultivars with a social, cultural, local or historical relation to Germany, and (3) cultivars with important pomological traits especially for breeders” [19]. For efficient conservation management, it is important to ensure cultivar authenticity. For this, extensive projects (funding codes: 2819BE003 and 2820BE003) were executed, in which the trueness-to-type of cultivars was assessed pomologically and molecularly. The cultivars in the GFG collection are majorly of the species *Pyrus communis*. Part of the GFG pear collection has already been studied in cooperation with Romania, but here a more holistic approach for the German collection is taken [21]. The GFG was able to follow similar approaches for their apple and sweet cherry collections as well [22,23]. In the study on apples, and for pears now as well, INRAE (National Research Institute for Agriculture, Food and the Environment, Paris, France) provided *Pyrus* UNiQue genotype codes (PUNQs) which enable harmonizing with more pear datasets in the future [24].

In this work, the published dataset of the GFG pear collection was used to investigate the genetic diversity, structure and parentage of the collection in varying contexts [25]. Five subsequent analyses are presented in this study: (I) the authenticity of the pear cultivars in the GFG collection, (II) the suitability of the used markers for subsequent analyses and the establishment of a suitable SSR marker set for variety authenticity testing, (III) an unbiased parentage analysis taking into account the literature information, (IV) the genetic structure on the background of cultivar origin, and (V) a genetic relationship analysis linked to the countries of origin of the individual pear cultivars.

## 2. Methods

The results and conclusions gained from this work are based on a previously published dataset and publicly available data provided by the German Fruit Genebank (GFG) [18,25]. On behalf of the German Federal Ministry of Agriculture, Food and Regional Identity, two projects (funding codes: 2819BE003 and 2820BE003) were set up between 2020 and 2023 to determine the authenticity (trueness-to-type) of cultivars preserved in the pear network of the GFG. In summary, 1945 pear samples were assessed pomologically and molecularly. After data review and compilation, 421 pear cultivars corresponding to individual genotypes were published and subsequently used in this work [25]. Sports of cultivars are classified under their originating cultivars and the base cultivar name. This is the case for seven cultivars with their respective sports, and by this process all used genotypes are unique. A detailed report on the methodology of the projects and the creation of the used dataset can be found in Broschewitz et al. (2026) [26].

**Figure 1:**
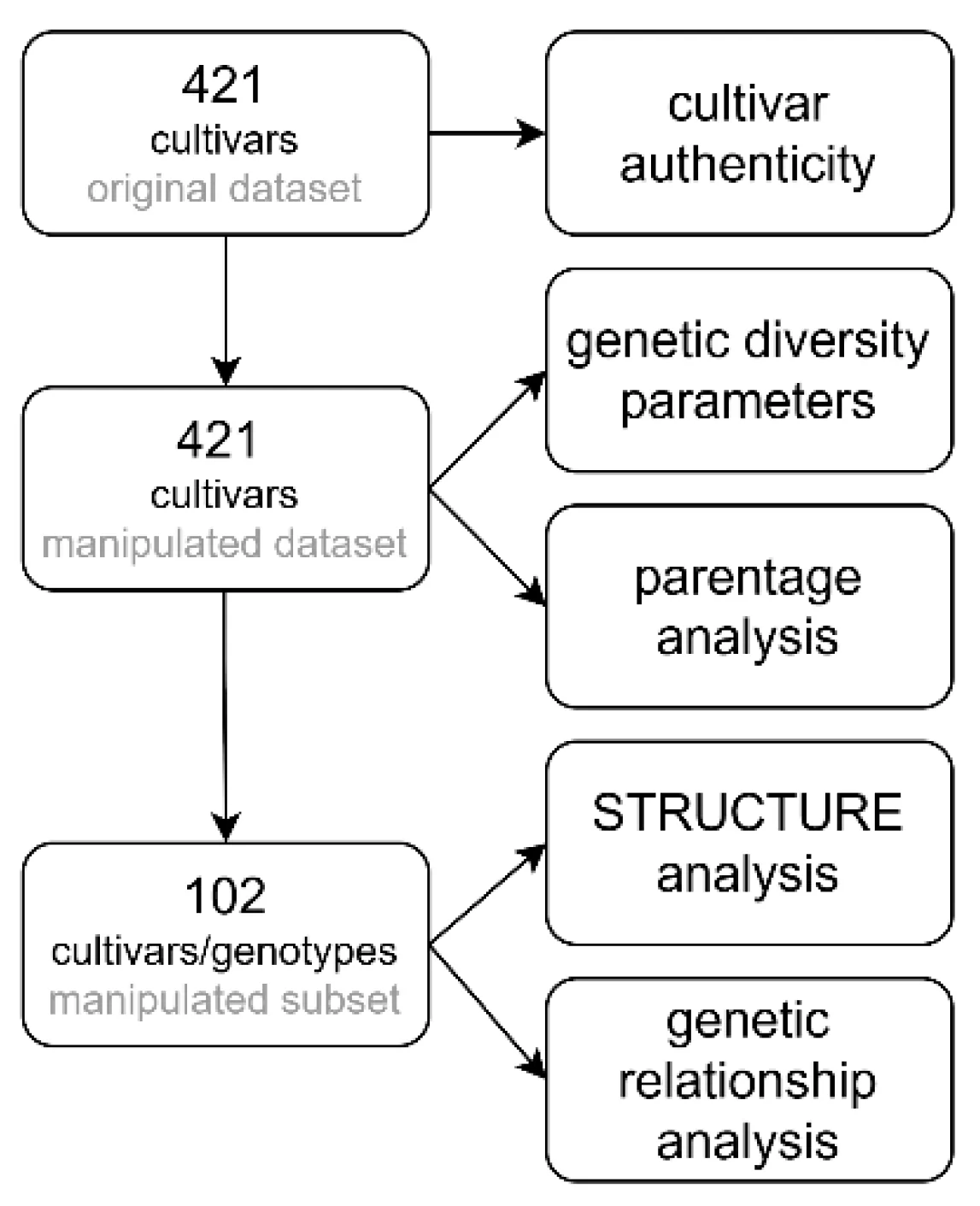
Flowchart on dataset use for specific analyses. Detailed manipulation of the dataset is described in dedicated sections.

In this study, the original dataset is used for the cultivar identification [25]. Here, no molecular data is needed. An overview of which data is used for which analysis is shown in Figure 1. The genetic diversity parameters and markers are assessed based on a manipulated dataset, still with 421 cultivars. The parentage analysis uses the same dataset. A subset of the manipulated dataset is used for the genetic structure and genetic relationship analysis. The detailed manipulation of the dataset is described in the concerned sections.

### 2.1. Trueness to Type and Cultivar Authenticity

The trueness-to-type criterion was established by the GFG as a standardized measure to determine the accuracy of cultivar identification. This criterion integrates both pomological and molecular datasets, with primary weight assigned to pomological evaluations. For the pear network, samples were assessed pomologically by Schlaraffenburger Streuobstagentur (Schlaraffenburger gGmbH, Aschaffenburg, Germany; https://schlaraffenburger.de/) and molecularly by Microsynth ecogenics GmbH (Balgach, Switzerland). For the molecular analysis, a set of 17 SSR markers based on the recommendation by the Malus/Pyrus Working group of the European Cooperative Programme for Plant Genetic Resources (ECPGR; http://www.ecpgr.cgiar.org/) were used [27]. The markers were originally mainly established in *Malus* spp., but were deemed suitable for *Pyrus* spp. [27,28,29,30,31,32,33]. Scores are assigned on a qualitative scale from “0” to “5”. In this dataset, categories “1” (true-to-type), “5” (true-to-type with reservation), “3” (not determined) and “4” (not assessed) are represented. Other categories include “2” (true-to-type, group/mutant) and “0” (deceased) for cultivars where no originating cultivar is within the collection or the cultivar was deceased from the collection after documentation. A comprehensive description of the scoring procedure is provided in the data descriptors by Broschewitz et al. (2026, 2025) [26,34]. Further on, categories “0”, “3” and “4” are generally referred to as not true-to-type. The trueness-to-type criteria for 421 cultivars are visualized based on the original dataset as no molecular data is needed [25].

### 2.2. Probability of Identity Calculation and Diversity Parameters

While obtaining the dataset for the following analyses, some adjustments were made to the original data [25]. This was necessary to account for requirements of the used bioinformatics tools. The bioinformatic tools are optimized for data with homogenous ploidy, preferably diploidy. As the original dataset was expected to contain mainly diploid cultivars, but several markers showed a multilocus behaviour, i.e., more than two allele variants as expected for diploids, and we also wanted to maintain the triploid cultivars, some adjustments had to be made. The level of ploidy per cultivar was inferred by flowcytometry through an internal investigation of the Julius Kühn Institute and published along with the original dataset [25]. Out of 421 pear cultivars, 92 (22%) were determined as diploid, 17 (4%) as triploid and 312 (74%) were not tested. In the manipulated dataset, only the first two allele variants are retained which coincides with the two smallest alleles. In a previous publication, the issue with excess allele variants is discussed and common genetic diversity parameters were used to assess the original data and manipulated subsets [35]. In the manipulated dataset, SSR markers CH01d09 and CH05a02 were omitted. Both markers showed up to six allele variants possibly due to stutter bands as well as highly overrepresented variants, possible artefacts, that were found throughout all genotypes. The two steps of manipulation, by allele variant and marker omission, influenced the genetic diversity parameters compared to the original dataset [35]. As the dataset was manipulated, original ploidy levels were artificially adjusted so that all cultivars are represented as diploid. The final analysis dataset contained 15 markers with two allele variants for 421 pear cultivars and is suitable for downstream analysis. The calculation of the diversity parameters and the probability of identity was performed with the R script SSR_DivParCal, version v1 [35,36].

### 2.3. Parentage Analysis

In CERVUS v3.0.7, a simulation for parent pairs of unknown sexes was run for 100,000 offspring and 450 candidate parents [37,38]. The number of candidate parents approximates the dataset size. Parameters were adjusted to the following settings: proportion (prop.) sampled 0.7, prop. loci typed 0.9826, prop. loci mistyped 0.01 and minimum typed loci 12. The prop. sampled was chosen as such because it is assumed that (historic) cultivars are missing from the collection. In the analysis, the 421 pear cultivars were tested against each other to infer the most likely offspring-parent trios from the given dataset. Upon the statistical evaluation of candidate parentages, filters for amount of loci mismatches and historical context were used to assess the results in detail. The emergence year of a cultivar was used to filter only for logical candidate parents that are at least 10 years older than their suggested offspring. The emergence year was gathered from the GFG and can represent the earliest year of crossing, mention or other based on the literature source. The 10 years are chosen arbitrarily.

### 2.4. Structure Analysis

The genetic structure was estimated with STRUCTURE v2.3.4 [39,40,41,42] and a specifically curated subset of data with 102 cultivars (Supplemental Table S1). The data subset was filtered for cultivars with complete records for emergence year and origin country Belgium, Germany or France. The data included information on the country of origin where it was restricted to Belgium (26 cultivars), Germany (34 cultivars) and France (42 cultivars) as there was a suitable number of cultivars for these available. This information was only used for graphs but not in the following calculations. The programme was run on default settings with 10,000 burn-in periods and 100,000 Markov Chain Monte Carlo repetitions. To assess the optimum number of genetic groups for the data, the program was set to 10 repetitions of each K, from K = 2 to K = 6. The runs were evaluated with the R package pophelper v2.3.1 using the Evanno-method [43,44]. With optimum K = 3 based on Evanno Delta K Method (see Supplemental Figure S1), the run with the highest mean value of ln likelihood was chosen for visualization. Between runs there was some variability between the clusters, but the chosen run represents the most consistent one where cultivars were put in the same cluster at least 6 out of 10 runs. Mean expected heterozygosity was calculated for the subset, each origin group, and each inferred cluster using GenAlEx v6.5 [45]. Additionally, pairwise fixation indices (F_ST_) were estimated separately among origin groups and among inferred clusters using AMOVA based on codominant allelic distances, which were assessed using default permutation test settings [45]. The information of emergence year was used in preliminary, inconclusive analyses and is not presented further in this work.

### 2.5. Analysis of Genetic Relationships

In DARwin v6.0.21 (Dissimilarity Analysis and Representation for Windows), a neighbour-joining dendrogram was built by calculating a dissimilarity matrix from allelic data (Supplemental Table S1) [46]. Missing data was processed by pairwise allele deletion for the minimal proportion of 90% of valid data required for each unit pair. A total of 1000 bootstraps were calculated. The unweighted neighbour-joining method was used to construct a tree representing genetic relationships among the pear genotypes. Then, in the web tool Interactive Tree of Life (iTOL) v6, labels and clades were coloured based on the country of origin [47].

## 3. Results

### 3.1. Authentic Cultivars in the Pear Network of the German Fruit Genebank

**Figure 2:**
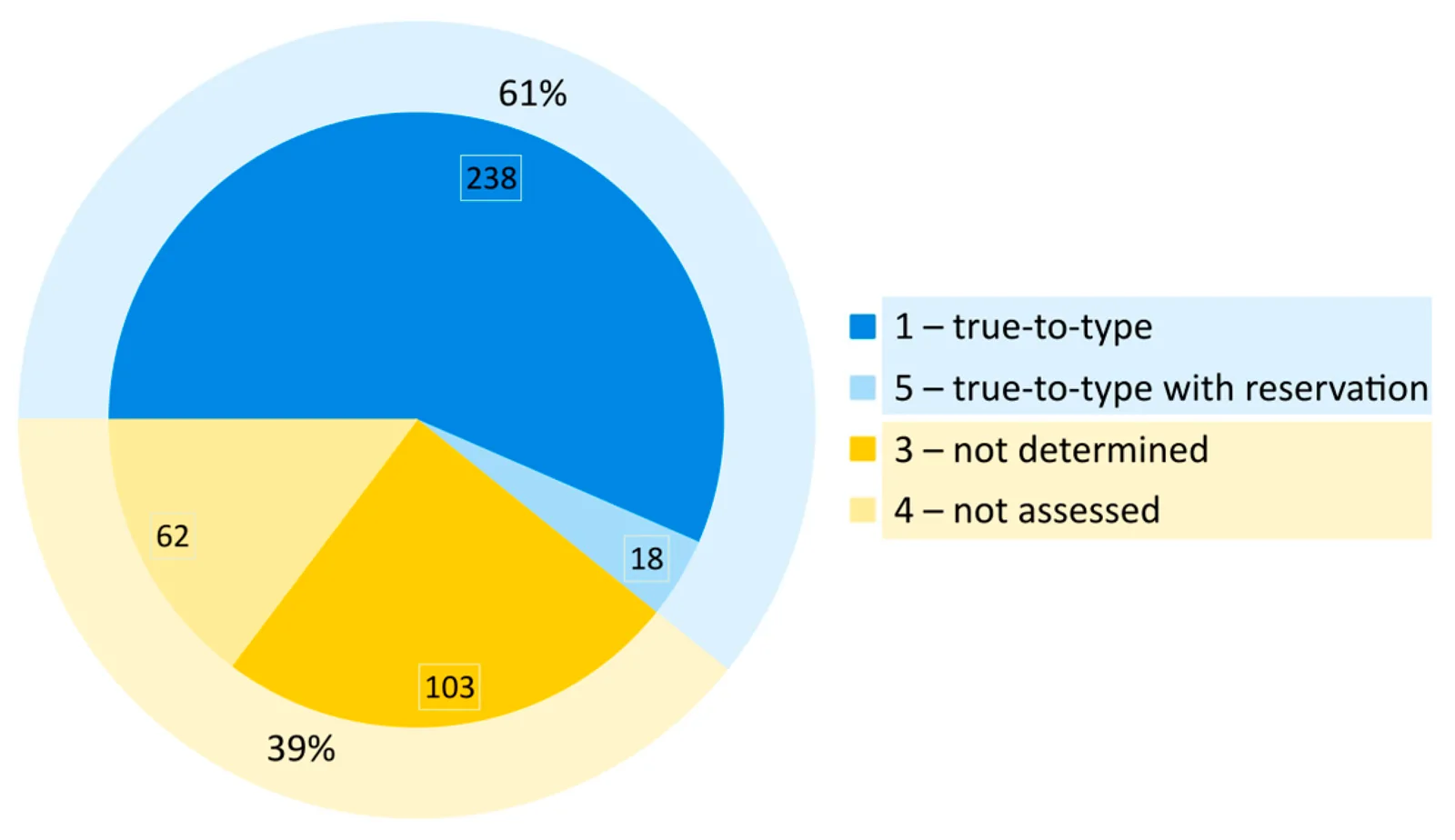
Representation of 421 pear genotypes corresponding to cultivars in the German Fruit Genebank (GFG) sectioned by authenticity of cultivar identification (trueness-to-type) given in percentage and count.

The pear collection of the GFG was assessed by assessing 1945 samples collected from eight partner institutions in Germany. After compiling the individual data to infer the amount of cultivars in the collection, 421 individual cultivar groups with unique genetic profiles were identified [25,26]. Seven of these cultivar groups include sports, which are pomologically distinguishable but molecularly identical and share the same genetic profile with their origin cultivar. The samples corresponded to trees that were assessed pomologically and tested molecularly. The pomological assessment was necessary to identify the cultivar name and trueness-to-type. The investigation showed that 61% of the cultivars had accessions which were true-to-type or true-to-type with reservation (Figure 2). Cultivars would be deemed not true-to-type when the pomologists did not know the cultivar, there were discrepancies in the references or there was no reference literature present. Not determined cultivars were pomologically assessed but identification was not possible. For not assessed cultivars the pomological assessment was not possible; only molecular data is available, and the cultivar identity remains uncertain.

### 3.2. Dataset Composition and Metrics of Markers

The dataset of all 421 genotype and 15 SSR markers showed a high genetic diversity as expressed by the mean expected heterozygosity mean *H_exp_* = 0.81 and mean number of alleles per locus mean *N_a_* = 20 (Table 1).

**Figure 3:**
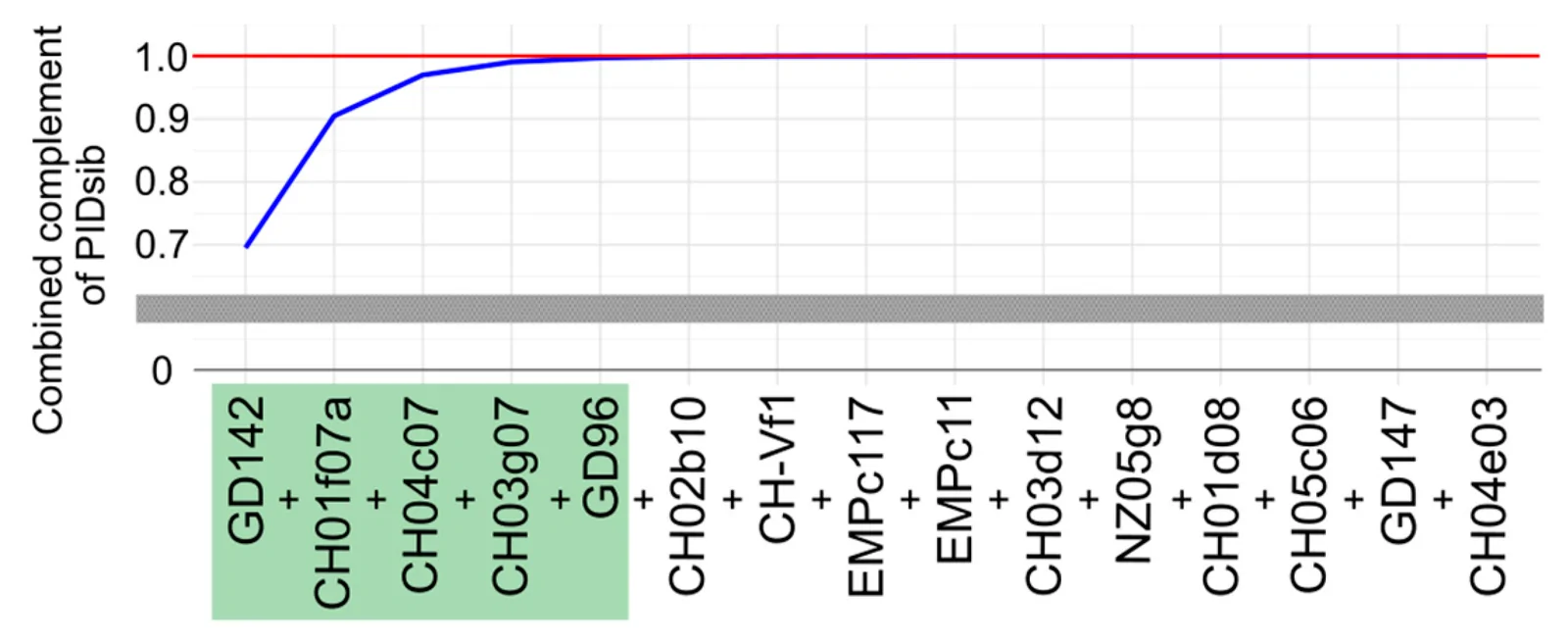
Combined effect of discrimination power of 15 SSR markers represented by the complement of average probability of identity of two siblings (PIDsib). The marker combination required for near 100% discrimination of the pear genotypes are highlighted in green. The red line marks the point at which the discrimination is 100 per cent. The blue line indicates the actual level of discrimination using the SSR markers shown on the x-axis.

The values for *N_a_* ranged from 30 different alleles for CH03g07 to 11 for CH04e03 and NZ05g8. The mean number of effective alleles (mean *N_e_*) was calculated to be much lower than mean *N_a_*, with mean *N_e_* = 6.43 and lowest *N_e_* = 2 (CH04e03) and highest *N_e_* = 9.97 (GD142). The two aforementioned markers CH04e03 and GD142 also represent the markers with the lowest and highest individual *H_exp_* of 0.5 and 0.9, respectively. Additionally, these two markers have the lowest and highest power of distinction between genotypes, based on their polymorphic information content PIC. The power of discrimination between genotypes was also expressed by the probability of identity of two unrelated individuals (PID) and probability of identity of two siblings (PIDsib). The smaller the values for these parameters are, the higher the power of discrimination. The cumulative effect of PIDsib can be observed in Figure 3. The markers GD142, CH01f07a, CH04c07, CH03g07, GD96 are highlighted as these indicate the threshold from which 99.5% of genotypes can be distinguished. With only this set of markers one should be able to confidently separate all 421 genotypes.

The highest discrepancy between *H_obs_* and *H_exp_* can be observed in marker CH04c07. For this marker, 23% of data is missing in the data, as seen in the number of assessed genotypes (Table 1). For most other markers, *H_obs_* and *H_exp_* are fairly similar; however, the disturbance of missing data is still recorded but with much less impact, i.e., in marker GD96 or CH03d12.

### 3.3. Parentage Analysis

In the parentage analysis, an unbiased approach was taken. We tried to identify direct relationships without prior knowledge and in the given dataset. The parentage information from the literature for the pear cultivars was very limited. So, all cultivars were treated as offspring and tested against all other genotypes to find possible parent pairs.

For 253 out of 421 offspring genotypes, candidate parent pairs with significant trio confidence were detected. However, even though the trio confidence for these suggested pairs were significant, some candidate pairs showed many loci mismatches. If the mismatches are additionally filtered to be less than 3, 113 reasonable matches for parent pairs are found (Supplemental Table S2). Including the information for emergence year by making sure the supposed offspring is at least 10 years younger than its candidate parents, only 19 suggested offspring-parent trios remain (Table 2). The number of results decreased drastically because of the lack of age information for the different cultivars.

For these 19 candidate offspring-parent trios, there was literature information on the parentage for seven of them (Supplemental Table S3). Votteler (1998) reported that ‘Condo’ is the offspring of ‘Conference’ and ‘Vereinsdechantsbirne’ and that the maternal parents for ‘König Karl von Württemberg’ and ‘Frau Luise Goethe’ are ‘Clairgeaus Butterbirne’ and ‘Esperens Bergamotte’, respectively [48]. This is in line with the findings of the CERVUS parentage analysis, but the programme does not distinguish between maternal and paternal parent in the investigated context. For four more cultivars the full parentage was described as follows: ‘Packhams Triumph’ (‘Uvedale St. Germain’ × ‘Williams Christ’), ‘Isolda’ (‘Dr. Jules Guyot’ × ‘Bunte Julibirne’), ‘Armida’ (‘Dr. Jules Guyot’ × ‘Vereinsdechantsbirne’) and ‘Eckehard’ (‘Nordhäuser Winterforelle’ × ‘Clapps Liebling’) [49]. Furthermore, the full sibship and half-sibship between ‘Eckehard’ and ‘Gerburg’ (‘Nordhäuser Winterforelle’ × ‘Clapps Liebling’), and ‘Eckehard’ or ‘Gerburg’ and ‘Graf Wilhelm’ (‘Vereinsdechantsbirne’ × ‘Nordhäuser Winterforelle’) was also previously reported [4].The parentages for ‘Isolda’, ‘Gerburg’, ‘Graf Wilhelm’ and ‘Eckehard’ were completely confirmed in this analysis (Table 2 and Table S2). For ‘Packhams Triumph’, ‘Williams Christ’ was also found as a parent but second parent is supposedly ‘Winternelis’ instead of the literature-based ‘Uvedale St. Germain’ [49]. In the case of ‘Armida’, both parents from the literature were in the dataset but the calculated parents differ completely (‘Frühe aus Trevoux’ × ‘Gräfin von Paris’, Table 2) [49].

### 3.4. Genetic Structure and Origin Country

For the investigation in the context of origin, a subset of the data was selected where both pieces of information were available. This subset included 102 cultivars. Then, an unbiased analysis was run to cluster the cultivars based on their genetic structure. The mean *H_exp_* of this subset of data was 0.76.

**Figure 4:**
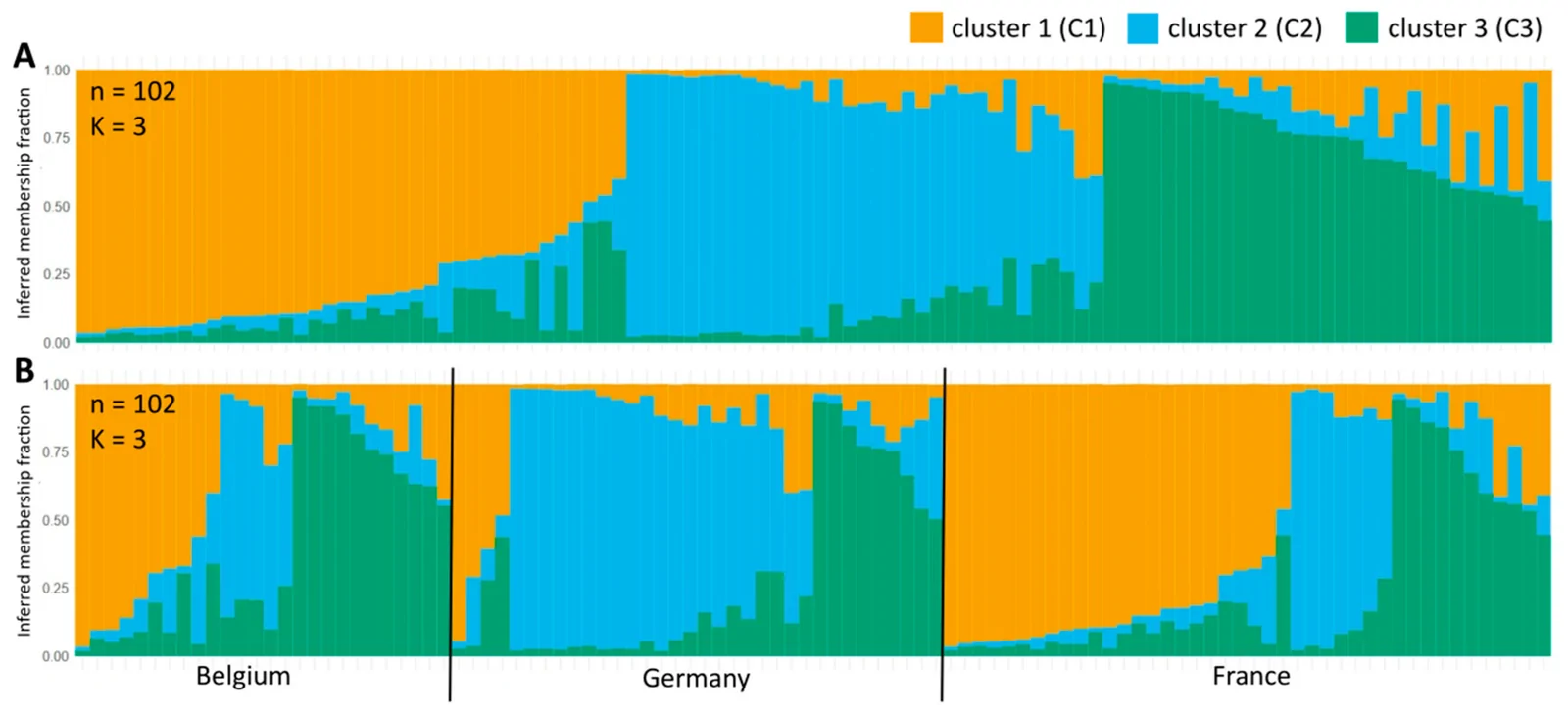
Genetic structure of 102 pear genotypes based on the STRUCTURE analysis of 15 SSR markers with optimum K = 3. Each genotype is represented by a vertical bar that is partitioned in colour according to the inferred membership fraction to clusters 1 to 3 (C1 to C3). (**A**) The cultivars are sorted by Q-value. (**B**) The cultivars are sorted by Q-value per origin group. For more detailed images with cultivar names, see Supplemental Figures S2 and S3.

The optimum number of clusters was determined to be three (K = 3; Supplemental Figure S1). When sorted by Q-value, the relatively even distribution of cultivars within each cluster is visible (Figure 4A and Figure S2). In the first cluster (C1), there are 38 cultivars, and 33 and 31 cultivars in the second (C2) and third cluster (C3), respectively. The mean *H_exp_* of the inferred clusters was 0.74, 0.77 and 0.77 for C1, C2 and C3, respectively. The distinction between the clusters was calculated as pairwise fixation index (F_ST_) and the highest value was 0.010 between C2 and C3. Pairwise F_ST_ values between C1 and C2 and between C1 and C3 were 0.004 and 0.006, respectively. Genetic differentiation, measured by pairwise F_ST_, was significant between C1 and C3 and between C2 and C3 (*p* < 0.05), but not between C1 and C2.

To add more context, the cultivars were sorted by country of origin. When the information on cultivar origin from Belgium, Germany or France is integrated, a slight shift in the cluster proportions is detected (Figure 4B and Figure S3). The genetic diversity of the origin groups seems comparable and less distinct than between the estimated clusters. The mean *H_exp_* for the three groups was calculated as 0.75, 0.77 and 0.77 for “Belgium”, “Germany” and “France”, respectively. The distinction between these groups is very low (F_ST_ ≤ 0.001). The genetic differentiation between either origin group variation was not significant (*p* < 0.05). In the “Germany” group the proportion of C2 is much larger compared to the other two groups. A total of 21 cultivars (ca. 62%) are primarily sorted in C2 that originated in Germany, while only five cultivars (ca. 19%) for Belgium and seven cultivars (ca. 17%) for France. The proportion of C1 is more prominent in the cultivars originating from France.

### 3.5. Genetic Relationships

**Figure 5:**
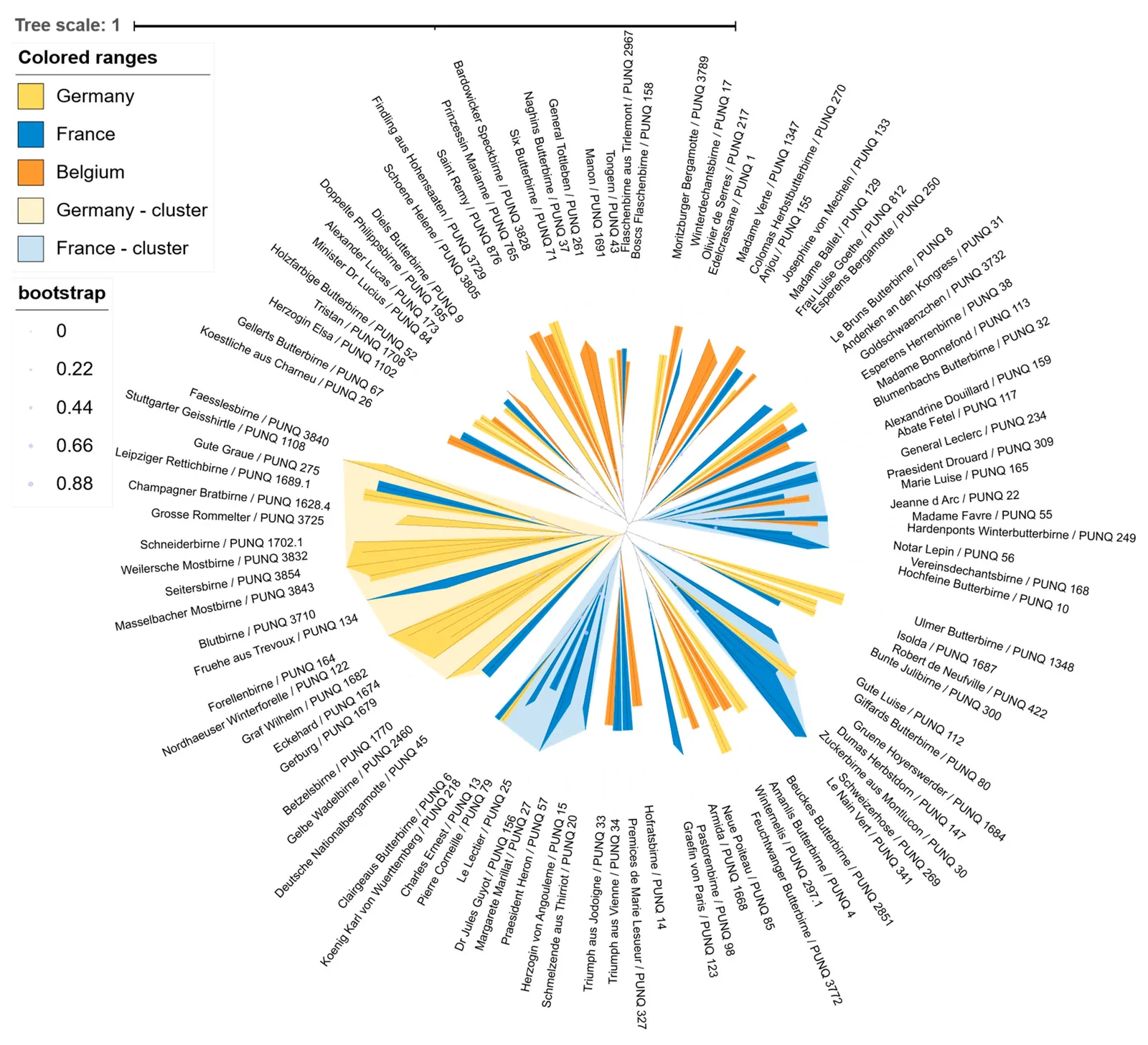
Neighbour-joining dendrogram of 102 pear cultivars with known country of origin. Country of origin is indicated by colour of the branches (yellow = Germany (DEU), blue = France (FRA), orange = Belgium (BEL)). Sub-clusters with cultivars that predominantly originated in Germany and France are highlighted in light yellow and light blue, respectively. ID labels follow the structure: “<cultivar name, special characters removed>/<PUNQ>”. This dendrogram was drawn with iTOL [47]. The size of the dots indicates the Bootstrap value (see also the legend in the figure).

This slight distinguishing trend based on the originating country can also be observed in the constructed tree representing genetic relationships among the 102 pear genotypes (Supplemental Table S1). In the unrooted tree, one can observe several branches, which mainly host cultivars from Germany and France, respectively (Figure 5). Specific sub-clusters can be explained by integrating the parentage analysis results as well (Supplemental Table S2).

In the predominantly German branch, there are several sub-branches that only include German cultivars. For example, there is the sub-branch comprising ‘Forellenbirne’, ‘Nordhäuser Winterforelle’, ‘Graf Wilhelm’, ‘Eckehard’ and ‘Gerburg’. The subsequent parentage analysis indicates that statistically ‘Nordhäuser Winterforelle’ could be a potential parent of the other four cultivars, making them siblings (Supplemental Table S2) [4]. In turn, this explains the close clustering based on similar genetic structure and genetic closeness. For the other German sub-branch which contains ‘Schneiderbirne’, ‘Weilersche Mostbirne’, ‘Seitersbirne’ and ‘Masselbacher Mostbirne’, the conclusion of close relatedness cannot be drawn as easily. The only significant result is ‘Schneiderbirne’ as a potential parent for ‘Masselbacher Mostbirne’. The direct relation between ‘Schneiderbirne’ and ‘Weilersche Mostbirne’ was not statistically confirmed, and for ‘Seitersbirne’, no connection to any of the other cultivars can be made so far.

Similarly difficult is the description of the French sub-branch with ‘Schweizerhose’, ‘Dumas Herbstdorn’, ‘Grüne Hoyerswerder’, ‘Zuckerbirne aus Montlucon’ and ‘Le Nain Vert’. Here, a significant and direct relation is suggested between ‘Schweizerhose’ and the other cultivars except for ‘Grüne Hoyerswerder’. The missing link between these two cultivars could be ‘Falsche Schweizer Hose’ (working title) which was not included in the genetic relationship analysis due to the lack of data on the origin country. ‘Falsche Schweizer Hose’ (working title) is suggested to be directly related with both ‘Schweizerhose’ and ‘Grüne Hoyerswerder’ (Supplemental Table S2).

## 4. Discussion

### 4.1. Gene Bank Management Relies on Authentic Cultivars

Maintaining a diverse pear germplasm is essential for future breeders for accessible genetic resources with important traits [50]. The role of managing this diversity in Germany is taken over by the GFG [51]. This discussed dataset of pear cultivars of the GFG comprises one of the biggest collections that is published to date. Even larger germplasm collections were reported by the U.S. [14,52] and Spain [17,53]. Other important pear producers such as Italy [15] and Portugal [16] also examined their respective collections; works for Tunisia [54,55] and a Romanian-German collaboration [21] were also published. Many more countries manage their own national collections worldwide [50]. A major advantage which sets this collection and the generated dataset apart from the others is the extensive study of the trueness-to-type. While the intentional or unintentional redundancy of accessions and cultivars in a collection can be assessed with only molecular analyses, cultivar authenticity is a more complicated issue. The phenotype examination by pomological experts is crucial to determine the authenticity and further understand the value of a collection. Only with certainty on the trueness-to-type of cultivars can the gene banks manage their resources for conservation and research accordingly and effectively. The GFG prides itself on having many cultivars classified as true-to-type or true-to-type with reservation. The assessment of cultivars by pomological experts is very time consuming and costly. Cultivars of the GFG collection that are categorized as not determined might only be authenticated by a different pomologist which knows that particular cultivar. Not assessed cultivars might be evaluated pomologically in the future. There are not many experienced pomological experts for pear and cultivar identification based on the literature is very uncertain. The phenotype of a tree can differ based on many factors such as the climate, weather and location. It is therefore important to build a reliable reference database to infer cultivars molecularly.

### 4.2. Comparison to Other Germplasm Collections

The genetic diversity of the collection in this study was deemed comparable to others [55,56]. The mean *H_exp_* is comparable to the study by Kajkut Zeljković et al. (2021) with mean *H_exp_* = 0.8, and somewhat higher than the one by Ouni et al. (2020), mean *H_exp_* = 0.622 [55,56]. However, direct comparison of different germplasm collections is difficult due to varying collection sizes and the use of different marker sets. The compared studies by Kajkut Zeljković et al. (2021) and Ouni et al. (2020) used 74 and 61 pear accessions, while this study contained 421 genotypes [55,56]. Another influencing factor may be the constitution of the collections. National germplasm collections focus on local cultivars and accessions, i.e., from Bosnia and Herzegovina or Tunisia. This becomes very apparent when trying to infer the most informative markers as well. Kajkut Zeljković et al. [56] made a similar graph to Figure 3 of this study. Their more informative markers, i.e., CH05c06, CH03d12 and GD147, only showed moderate to low importance in this study. Next to the dataset size and different marker set, Kajkut Zeljković et al. [56]) plotted PID while this study used PIDsib as we suspect closely related individuals in the GFG collection, and PIDsib accounts for this.

### 4.3. Candidate Marker Panel for Pear Cultivar Identification

For this dataset of 421 genotypes, the five markers GD142, CH01f07a, CH04c07, CH03g07 and GD96 showed estimated cumulative discrimination probability of approximately 99.5% under statistical assumption. By leaving out CH04c07, as this marker showed a lot of missing data, the other four markers showed an estimated cumulative discrimination probability of approximately 99%. The high discrepancy between the mean *H_obs_* and mean *H_exp_* in CH04c07 can be explained by the missing values but could also be due to heterozygote deficit. Nevertheless, the marker still shows high allelic diversity and polymorphic information content which supports its use. For the GFG collection, it may be suitable to create a customized multiplex with these four or five markers to identify further accessions with minimal resources for routine cultivar identification. The fluorophores would need adjustment from the originally used combinations to guarantee proper results [26]. This subset of markers would be highly sensitive to missing data and technical errors or shifts. Overall, we propose an efficient candidate panel of markers that still need validation across other cultivars, gene banks and laboratories.

### 4.4. The Use of PUNQs for Harmonizing Data in the Future

An important and unique point of this work is the use of the internationally harmonized PUNQs [24]. While this system was previously utilized for apple, the use for pear has not been reported to our knowledge so far. The use of PUNQs instead of or in combination with cultivar names ensures clear alignment of data and conclusions in comparative studies. A common issue in the works on cultivars is the use of synonym and homonyms or location specific names and translations. By harmonizing the data with the PUNQs, it is clear which genotype is meant despite the use of a variety of different accession and cultivar names. However, when assigning the initial PUNQ number, it would be important that the variety was also determined pomologically. The ECPGR also puts a lot of effort into expanding their genotyped collection from a variety of different countries [57]. Therefore, we think that PUNQs will gain more relevance in the pomology and molecular biology community of pear in the future.

### 4.5. Parentage Analysis

The unbiased parentage analysis of 421 cultivars yielded 19 candidate offspring-parent trios while limiting the mismatched loci and including age information for logical comprehension. This filtering process was very restrictive, and even then, there were contradictory results with respect to the available literature, i.e., in the cases of ‘Packhams Triumph’ and ‘Armida’. For ‘Packhams Triumph’, the literature suggests “Uvedale St. Germain” as a parent [49]. Here, the issue might be that “Uvedale St. Germain” is not part of the tested dataset. ‘Winternelis’ and ‘Uvedale St. Germain’ might be closely related and, therefore, ‘Winternelis’ was suggested as a candidate parent. In this case, it was shown that missing genotypes could possibly lead to wrongful conclusions, which is one of the difficulties of unbiased analyses. Parentage analysis can also be performed for only the suggested literature data, but a lot of information will be lost, especially in the case of pear where there is not much parentage information available in the literature. For “Armida”, a completely different set of candidate parents was inferred, despite the availability of the literature-based parent genotypes. Based on a visual assessment of the allele variants, the candidate parentage of this study seems more likely. It might be that the authenticity of either cultivar is not correct, resulting in such an error. The process of gathering the literature information is tedious and time consuming, and while the GFG tries to incorporate this in their website, there is also a lot of misinformation and contradiction. Overall, this parentage analysis showed that there are a lot of factors to consider, and that it is very difficult to label a very certain parentage.

### 4.6. Genetic Structure and Genetic Relationships

The genetic structure analysis revealed that, when the cultivars are ordered per origin country, for Germany, cluster 2 constitutes the majority of cultivars, and for France, this is observed with cluster 1. Nevertheless, no significant distinction was noted among these two origin groups. The genetic differentiation between estimated clusters was significant between the pairs of cluster 1 and 3, as well as cluster 2 and 3. The observed genetic structure did not clearly correspond to the recorded geographic origins of the pear cultivars. Further investigation incorporating pedigree information and historical records of cultivar breeding and exchange is needed to clarify the processes underlying the observed genetic differentiation among clusters. The dendrogram showed clusters which consist of mainly German and French cultivars. For Belgium, we could not find such distinct branches in the tree. In the dendrogram, the cultivars of one of the highlighted German sub-branches, ‘Graf Wilhelm’, ‘Eckehard’ and ‘Gerburg’, are mentioned by Fischer (2005) as resulting cultivars of a breeding programme in Pillnitz/Naumburg known under the umbrella term Saxonia^TM^ [4]. Modern breeding can influence the genetic structure. France has patented several cultivars over the years, while there was not much information on Belgium. The conscious introduction of certain genotypes by dedicated breeding could then explain the specific formation of cluster constitutions for German and French pear cultivars but not Belgian ones. The analysis for genetic structure and for genetic relationships visualize the SSR variation in the same dataset and cannot be seen as independent validation of the discussed influence of origin country in pear cultivars. The drawn conclusions might not be applicable for the extended dataset or a different pear collection. While the statistical evidence for a clear genetic structure is lacking, a trend can be visualized which might be interesting to investigate with a different pear collection.

### 4.7. Limitations of This Study

This study showed a variety of analyses, but these come with different limitations. The used dataset was manipulated in terms of the number of retained alleles. More and different alleles and allele dosages will influence the outcomes presented here. The implication of ploidy was disregarded in this study and exchanged for the use of specific bioinformatic tools. In order to use it, a suggested candidate panel of markers for cultivar identification needs to be validated. In the STRUCTURE analysis and the neighbour-joining dendrogram, only a subset of data was used which might not be representative for the complete dataset. In the parentage analysis, candidate parents were possibly missing. Furthermore, the available literature information can be incomplete, uncertain or erroneous which decreases its reliability, i.e., the emergence age or reported candidate parents. These errors are very difficult to account for. While this work shows interesting results and directions for further studies, there are many limitations which this study tries to make as transparent as possible.

## 5. Conclusions

The pear is an important fruit for consumption with a longstanding history in Germany, Europe and worldwide. In the introduction, the current literature on pear genetic resources was summarized and put into context with the current study. We highlighted the importance of the work of the GFG and the necessity of cultivar authenticity. With this study, we evaluated the GFG collection and presented a range of analyses of the SSR data, including parentage, genetic structure and genetic relation.

The display of authenticity of the pear cultivars of the GFG showed that the collection is high-quality with a total of 421 different cultivars with unique genotypes, 61% of which have accessions that are true-to-type or true-to-type with reservation. With the used markers and SSR results, we could also show that a minimal marker panel could be suitable for cultivar distinguishment after reliable validation. A parentage analysis was performed where 19 candidate offspring-parent trios were identified and discussed with the available literature. The genetic structure of a subset of cultivars was investigated in the context of cultivar origin. The subset was grouped into three clusters. Genetic differentiation among some inferred clusters, but not among origin groups, suggests that the observed genetic structure is not clearly associated with the recorded geographic origins of the pear cultivars. Lastly, the dendrogram based on genetic relationships picked up the trend from the genetic structure analysis where specific sub-branches for German and French cultivars were identified. The formation of these sub-branches was discussed in the context of parentage and prior breeding programmes. The analysis of the genetic structure and the neighbour-joining dendrogram shows consistent visualization of SSR variation in the used dataset. Further analyses, with more extensive and different data, will be insightful.

With the aforementioned analyses, we presented a range of applications for the GFG pear cultivar SSR data [25]. The valuable GFG collection is an important treasure of genetic resources for future breeding. As there is market demand but a lack of breeding projects in Germany currently, we predict that pear breeding will become more relevant.

## Figures and Tables

**Table 1:** Genetic parameters of 15 SSR markers used in the molecular characterization of 421 pear cultivars of the GFG.

Locus	Assessed Genotypes	*N_a_* *	*N_e_* *	*H_obs_* * (Locus)	*H_exp_* * (Locus)	PIC *	PID *	PIDsib *
CH04c07	323	26	8.25	0.27	0.88	0.87	0.02	0.32
CH02b10	420	21	7.60	0.68	0.87	0.86	0.03	0.32
CH05c06	420	19	4.54	0.84	0.78	0.75	0.08	0.38
EMPc117	421	23	7.30	0.82	0.86	0.85	0.03	0.33
GD96	417	20	8.06	0.68	0.88	0.86	0.03	0.32
EMPc11	421	14	6.31	0.85	0.84	0.83	0.04	0.34
GD142	421	24	9.97	0.91	0.90	0.89	0.02	0.30
GD147	421	17	2.88	0.68	0.65	0.63	0.15	0.46
CH01d08	420	16	4.78	0.82	0.79	0.76	0.07	0.37
CH03g07	419	30	8.25	0.90	0.88	0.87	0.03	0.32
CH01f07a	421	25	8.81	0.86	0.89	0.88	0.02	0.31
CH-Vf1	421	19	7.48	0.82	0.87	0.85	0.03	0.32
NZ05g8	421	11	4.84	0.83	0.79	0.76	0.07	0.37
CH04e03	421	11	2.00	0.52	0.50	0.48	0.27	0.57
CH03d12	418	24	5.36	0.69	0.81	0.79	0.06	0.36
Mean	413.7	20	6.43	0.74	0.81	0.80	0.06	0.36

* *N_a_*: number of different alleles; *N_e_*: number of effective alleles; *H_obs_*: observed heterozygosity; *H_exp_*: expected heterozygosity, PIC: polymorphic information content, PID: average probability of identity of two unrelated individuals, PIDsib: average probability of identity of two siblings.

**Table 2:** A total of 19 offspring-parent trios with consistency in trio-confidence (95%), less than three mismatches and year information. For each offspring, parent 1 and parent 2, the cultivar name, PUNQ and emergence year is provided. The complete result table can be viewed in Supplemental Table S2.

Cultivar Name Offspring	PUNQ Offspring *	Year Offspring	Cultivar Name Parent 1	PUNQ Parent 1 *	Year Parent 1	Cultivar Name Parent 2	PUNQ Parent 2 *	Year Parent 2
Frühe aus Trevoux	134	1862	Williams Christ	24; 24.1	1770	Holzfarbige Butterbirne	52	1810
Condo	518; 518.4	1965	Vereinsdechantsbirne	168	1849	Conference	131	1884
Le Bruns Butterbirne	8; 8.1	1862	Williams Christ	24; 24.1	1770	Hardenponts Winterbutterbirne	249	1759
Flaschenbirne aus Tirlemont	2967	1880	Boscs Flaschenbirne	158; 158.1	1807	Pitmaston	35; 35.1; 35.4	1841
König Karl von Württemberg	218; 218.1	1886	Clairgeaus Butterbirne	6; 6.1; 6.4	1848	Herzogin von Angouleme	15	1809
Frau Luise Goethe	812	1882	Josephine von Mecheln	133	1830	Esperens Bergamotte	250; 250.1	1830
Notar Lepin	56	1879	Herzogin von Angouleme	15	1809	Hardenponts Winterbutterbirne	249	1759
Le Lectier	25; 25.1	1882	Clairgeaus Butterbirne	6; 6.1; 6.4	1848	Hardenponts Winterbutterbirne	249	1759
Packhams Triumph	476	1896	Williams Christ	24; 24.1	1770	Winternelis	297.1	1813
Präsident Drouard	309; 309.1	1886	Herzogin von Angouleme	15	1809	Hardenponts Winterbutterbirne	249	1759
Dicolor	3807	2006	Schmelzende aus Thirriot	20	1858	Nordhäuser Winterforelle	122; 122.1	1864
Gerburg	1679	2002	Clapps Liebling	118	1860	Nordhäuser Winterforelle	122; 122.1	1864
Tristan	1708; 1708.1	1988	Bunte Julibirne	300	1857	Clapps Liebling	118	1860
Isolda	1687	1995	Bunte Julibirne	300	1857	Dr. Jules Guyot	156; 156.4	1875
Manon	1691	2001	Clapps Liebling	118	1860	Boscs Flaschenbirne	158; 158.1	1807
Armida	1668	1961	Frühe aus Trevoux	134	1862	Gräfin von Paris	123; 123.1	1882
Eckehard	1674	1998	Clapps Liebling	118	1860	Nordhäuser Winterforelle	122; 122.1	1864
Graf Wilhelm	1682; 1682.1	2002	Vereinsdechantsbirne	168	1849	Nordhäuser Winterforelle	122; 122.1	1864
Superbia	690	1987	Williams Christ	24; 24.1	1770	Vereinsdechantsbirne	168	1849

* integer value = regular PUNQ attributed without doubts; value with suffix “.1” = some doubt about the PUNQ attribution due to small differences in SSR genotypes of the compared accessions; value with suffix “.4” = more than 3 discrepancies. For further explanation refer to sources [25,26].

## Data Availability

The original contributions presented in this study are included in the article/Supplementary Materials. The source dataset repository can be accessed via doi:10.5073/20250814-135212-0 [25]. Further inquiries can be directed to the corresponding author.

## Supplementary Materials

The following supporting information can be downloaded at: https://www.mdpi.com/article/10.3390/plants15213385/s1, Supplemental Table S1: Analysis dataset, subset of doi:10.5073/20250814-135212-0 for STRUCTURE analysis and neighbor-joining dendrogram construction with information for the origin country. Supplemental Table S2: Unbiased parentage analysis for both unknown parents. Information for PUNQ, trueness-to-type, country of origin and emergence year was added after the analysis for context. Supplemental Table S3: Literature check of 19 cultivars with confident results in parentage analysis (Supplemental Table S2). Supplemental Figure S1: Delta K of the STRUCTURE initial results with the Evanno Method to determine the optimum number of clusters (K). Supplemental Figure S2: Genetic structure of 102 pear genotypes based on the STRUCTURE analysis of 15 SSR markers with optimum K = 3, sorted by Q. Supplemental Figure S3: Genetic structure of 102 pear genotypes based on the STRUCTURE analysis of 15 SSR markers with optimum K = 3, sorted by cultivar origin.
